# Real-World Outcomes of Patients With Malignant Pleural Mesothelioma Receiving a Combination of Ipilimumab and Nivolumab as First- or Later-Line Treatment

**DOI:** 10.1016/j.jtocrr.2024.100735

**Published:** 2024-10-15

**Authors:** Sabine Schmid, Lisa Holer, Katrin Gysel, Kira-Lee Koster, Sacha I. Rothschild, Laura A. Boos, Lorenz Frehner, Sabine Cardoso Almeida, Christian Britschgi, Yannis Metaxas, Michael Mark, Patrizia Froesch, Wolf-Dieter Janthur, Anna Allemann, Christine Waibel, Catherine Von der Mühll-Schill, Martin Früh, Laetitia A. Mauti

**Affiliations:** a Department of Medical Oncology, Inselspital, Bern University Hospital, University of Bern, Bern, Switzerland; b Swiss Group for Clinical Cancer Research (SAKK), Bern, Switzerland; c Department of Medical Oncology and Hematology, Cantonal Hospital St. Gallen, St. Gallen, Switzerland; d Department of Medical Oncology, University Hospital Basel, Basel, Switzerland; e Department of Oncology/Hematology, Cantonal Hospital Baden, Baden, Switzerland; f Department of Medical Oncology and Hematology, University Hospital Zürich, Zürich, Switzerland; g Department of Medical Oncology and Hematology, Cantonal Hospital Winterthur, Winterthur, Switzerland; h Department of Medical Oncology, Cantonal Hospital Münsterlingen, Münsterlingen, Switzerland; i Department of Medical Oncology, Cantonal Hospital Chur, Chur, Switzerland; j Università della Svizzera Italiana, Lugano, Switzerland; k Department of Medical Oncology, Istituto Oncologico della Svizzera Italiana, Ente Ospedaliero Cantonale, Bellinzona, Switzerland; l Department of Medical Oncology, Cantonal Hospital Aarau, Aarau, Switzerland; m Department of Medical Oncology, Cantonal Hospital Lucerne, Lucerne, Switzerland; n Department of Medical Oncology, Claraspital Basel, Basel, Switzerland

**Keywords:** Malignant pleural mesothelioma, Ipilimumab, Nivolumab, Real-world, Outcome

## Abstract

**Objectives:**

On the basis of the positive results of CheckMate-743, first-line (1L) treatment of malignant pleural mesothelioma (MPM) with the combination of ipilimumab and nivolumab (ipi-nivo) has become a standard-of-care. Furthermore, patients who received 1L platinum/pemetrexed chemotherapy are often considered for second or further-line (2L+) ipi-nivo on the basis of MAPS2 results. Here we report on real-world survival and safety outcomes of ipi-nivo for patients with MPM in Switzerland.

**Methods:**

Twelve cancer centers contributed data to this retrospective cohort. Baseline characteristics including age, sex, histology, programmed death-ligand 1 expression, Eastern Cooperative Oncology Group performance status (ECOG PS), and previous/subsequent therapies were collected. The efficacy and safety outcomes were assessed by local investigators.

**Results:**

Of the 109 patients with MPM treated with ipi-nivo between November 2017 and March 2023 (median follow-up of 16.6 months) 75% had epithelioid, 9% biphasic, and 16% sarcomatoid histology. The median age was 72 years; 91% were males, and 83% had an ECOG PS of 0 to 1. Ipilimumab in combination with nivolumab was given as 1L in 43% and as 2L+ treatment in 57% of patients. The objective response rate was 21% in 1L and 15% in 2L+. Median progression-free survival was 6.5 and 2.8 months, respectively. Median overall survival (OS) from the start of ipi-nivo was 12.6 months for 1L and 6.9 months for 2L+. No differences in OS were observed depending on age and programmed death-ligand 1 expression. Nevertheless, the median OS was significantly worse in patients with an ECOG PS of 2 or higher than those with an ECOG PS of 0 to 1 (2.4 versus 11.9 months, *p* < 0.001). Treatment-related adverse events (TRAEs) of any grade related to ipi-nivo treatment occurred in 65 patients (62%). The highest-grade TRAE was 1 to 2 in 58% of these patients and 3 or higher in 42% Treatment discontinuation due to a TRAE occurred in 22% of patients.

**Conclusion:**

In this real-world cohort of patients with MPM treated with ipi-nivo survival outcomes were inferior to those reported in the CheckMate-743 and MAPS2 trials, whereas safety outcomes were similar.

## Introduction

Combined immune checkpoint inhibition with ipilimumab and nivolumab (ipi-nivo) has become a standard-of-care first-line (1L) treatment of malignant pleural mesothelioma (MPM) on the basis of the randomized phase III CheckMate-743 (CM743) trial.^1^^,^^2^ A significant and clinically relevant improvement in median overall survival (OS) of 18.1 versus 14.1 months (hazard ratio [HR] = 0.74, *p* = 0.002) was observed in the overall population treated with ipi-nivo compared with platinum/pemetrexed chemotherapy with a pronounced benefit in patients with non-epithelioid histology (median OS: 18.1 versus 8.8 months, HR = 0.46).^1^ Positive programmed death-ligand 1 (PD-L1) expression (≥1% of tumor cells) also correlated with better outcomes (HR = 0.71, 95% confidence interval [CI]: 0.57–0.88). On the basis of these subgroup analyses, ipi-nivo has been approved in Switzerland for the 1L treatment of non-epithelioid MPM and epithelioid MPM with a PD-L1 expression of 1% or higher. This is in contrast to the Food and Drug Administration and the European Medicines Agency labels that are irrespective of histology and PD-L1 status. Nevertheless, for patients with epithelioid mesothelioma and unknown or negative PD-L1 expression off-label reimbursement for 1L ipi-nivo could be obtained through a special request either before combination ipi-nivo was approved for MPM or because patients had a contraindication to 1L chemotherapy.

Grade 3 and 4 treatment-related adverse events (TRAEs) occurred in 30% of the 303 patients treated with ipi-nivo, with the most common higher-grade immune-related adverse events (AEs) being diarrhea/colitis (n = 12), hepatitis (n = 14), rash (n = 8), pneumonitis (n = 6), and nephritis (n = 5). Three treatment-related deaths occurred in the ipi-nivo arm (pneumonitis, encephalitis, and acute heart failure). Overall, 52 patients discontinued ipi-nivo due to immune-related AEs with a median treatment duration of 4.3 months. Survival outcomes in these patients were comparable to the overall cohort with a median OS of 25.4 months.^2^

Until recently, there was no standard of care second-line (2L) treatment for MPM after failure of platinum-based chemotherapy, and the treatment options of single-agent vinorelbine or gemcitabine were mostly on the basis of small single-arm phase II trials with modest results.^3^ Single-agent vinorelbine in combination with active symptom control c a significant improvement in progression-free survival (PFS) versus active symptom control alone, yet this did not translate into a significant improvement in OS.^4^ In contrast, in the randomized phase II RAMES combination of gemcitabine/ramucirumab led to a significant OS-benefit compared with treatment with gemcitabine alone (13.8 versus 7.5 months, HR 0.71, *p* = 0.028)^5^; nevertheless, confirmatory phase III trials so far are lacking. The phase II PROMISE-Meso trial failed to report a PFS benefit of 2L pembrolizumab versus the investigator's choice single-agent chemotherapy (gemcitabine or vinorelbine),^6^ whereas the randomized placebo-controlled CONFIRM trial reported a survival benefit for patients receiving second-line nivolumab (median OS: 10.2 versus 6.9 months, HR = 0.69, *p* = 0.009).^7^ In addition, the randomized non-comparative two-arm MAPS2 trial found promising efficacy outcomes in patients receiving second or further-line (2L+) treatment with nivo (n = 63) or ipi-nivo (n = 62) at the same dose of nivo 3 mg/kg every two weeks and ipi 1 mg/kg every six weeks as in CM743.^8^ The primary endpoint of 12-week disease control rate (DCR) was reached in 44% of patients with nivo alone and 50% with ipi-nivo. With the combination treatment an objective response rate (ORR) of 31%, a median PFS of 5.6 months, and a median OS of 15.9 months were observed. The rate of grade 3 to 4 AEs in this highly selected phase II patient population was 14% with nivo and 26% with ipi-nivo, including 3 treatment-related deaths (5%) in the latter group (hepatitis, encephalitis, and acute kidney failure). On the basis of the results of the MAPS2 trial, some patients in Switzerland have been receiving off-label 2L or 2L+ treatment with ipi-nivo after failure of platinum/pemetrexed (±bevacizumab).

As with most registrational trials, only a small percentage of older patients defined as 75 years of age or older (26%, n = 78) were randomized to ipi-nivo in the CM743 trial, and patients with an Eastern Cooperative Oncology Group performance status (ECOG PS) of 2 or higher were excluded in CM743 and MAPS2. In the subgroup analysis of patients aged 75 years or older in CM743, no benefit for ipi-nivo was observed (HR for OS = 1.02). Yet 60% of patients with MPM in Switzerland are 70 years or older at the time of diagnosis. We hypothesized that in a real-world (RW) setting including older patients or patients with a poor ECOG PS survival and toxicity outcomes with ipi-nivo might differ from those reported in a trial population.

The primary objectives of this study were to evaluate the efficacy and safety of ipi-nivo in an RW population of patients with MPM in Switzerland in first and later-line settings and to investigate potential prognostic clinical biomarkers for response to ipi-nivo in this population.

## Materials and Methods

### Patient Population

Patients with MPM who received a combination ipi-nivo in any line of treatment between November 2017 and March 2023 at 12 cancer centers in Switzerland were included in this analysis. Clinical, radiological, and pathological data were collected by local investigators.

### Assessments and Data Collection

Patients must have received adequate follow-up including documented radiological evaluation of tumor manifestations generally with computed tomography every two to three months. Anonymized data was entered into a central electronic database at the Swiss Group for Clinical Cancer Research Competence Center. Data on patient and tumor characteristics (including age, gender, performance status, history of atopy/allergy (as defined by local investigator), tumor stage, and histological subtype) and treatment details for ipi-nivo and any other systemic or local treatments were collected. The Charlson Comorbidity Index (CCI) was calculated as described originally.^9^ Tumor assessments were performed by local radiologists mostly using the revised modified Response Evaluation Criteria in Solid Tumors version 1.1 criteria for MPM.^10^ Best overall response was recorded alongside survival status and immune-related adverse events and their treatment. Treatment beyond progression was defined using a 21-day cut-off (treatment continued at least >21 days after radiographic disease progression). Where assessed by local pathology, results of PD-L1 and BRCA1-associated protein 1 (BAP1) staining and CDKN2/p16 assessment by fluorescence in situ hybridization or immunohistochemistry were also collected. Programmed death-ligand 1 expression on tumor cells was assessed by the local pathologists applying their standard clones and platforms (SP263, 73-10 in most of the cases). BRCA1-associated protein 1 staining and CDKN2/p16 assessment by fluorescence in situ hybridization or IHC was also performed by local pathologists and details on methods/clones used were not collected.

Approval from the Ethics Committee of Zürich (Lead Ethics), Eastern Switzerland, Northern Switzerland, Ticino, and Bern was obtained before starting data collection (BASEC Nr. 2022-01537). Most of the patients signed a general consent for further use of their data and material. For those patients who had passed or could not be contacted, we obtained an exemption according to Article 34 of the Human Research Act to use the data without explicit consent to study participation from the ethics committees in Switzerland provided there was no record of objection to further use of personal and disease-related data.

### Statistical Analysis

The endpoints are defined in detail in the supplementary material. For time-to-event endpoints, the median and the 95% CI were calculated using the Kaplan-Meier method. Differences in time-to-event outcomes between groups of patients were assessed using the log-rank test. To compare proportions and continuous variables between groups of patients Fisher’s exact test and Kruskal Wallis test were used respectively. There was no control for multiple testing. All statistical analyses were performed using the SAS software version 9.4 and R version 4.1.2.

## Results

### Patient and Tumor Characteristics

The patient and tumor characteristics have been reported in Table 1. Until the data cut-off on May 1, 2023, 109 patients had been included in this registry. The median age was 72 (range, 49-87) with 45 (41%) patients aged 75 years or older. The majority were males (91%), and had a history of asbestos exposure (86%). A total of 17 patients (17%) had an ECOG PS of 2 or higher at the start of ipi-nivo. Meanwhile, 58 patients (53%) had a CCI score of 6 or higher (70% patients starting 1L ipi-nivo and 40% patients starting 2L+ ipi-nivo), with higher values indicating more comorbidities. Twenty-three patients (21%) had undergone decortication/pleurectomy or extended pleuro-pneumonectomy (EPP)—all of them before receiving ipi-nivo.

**Table 1:** Baseline Characteristics

Variable	Category	Overall Cohort	Ipi-Nivo First Line	Ipi-Nivo Second or Later Line
Total N (%)		109 (100)	47 (100)	62 (100)
Sex	Female Male	10 (9) 99 (91)	3 (6) 44 (94)	7 (11) 55 (89)
Age at diagnosis	Median (range)	72 (49-87)	77 (62-86)	68 (49-87)
Age at start ipi-nivo	Median (range) <75 years ≥75 years	72 (55-93) 64 (59) 45 (41)	77 (62-88) 16 (34) 31 (66)	69 (55-93) 48 (77) 14 (23)
ECOG PS at start ipi-nivo	0 1 ≥2 Missing	24 (24) 58 (59) 17 (17) 10	14 (31) 26 (58) 5 (11) 2	10 (19) 32 (59) 12 (22) 8
CCI	<6 ≥6	51 (47) 58 (53)	14 (30) 33 (70)	37 (60) 25 (40)
Asbestos exposure	Yes No Missing	79 (86) 13 (14) 17	30 (79) 8 (21) 9	49 (91) 5 (9) 8
Histology	Epithelioid Biphasic Sarcomatoid	82 (75) 10 (9) 17 (16)	28 (60) 7 (15) 12 (25)	54 (87) 3 (5) 5 (8)
PD-L1 (TPS)	<1% ≥1% Missing	37 (64) 21 (36) 51	15 (56) 12 (44) 20	22 (71) 9 (29) 31
BAP-1 (IHC)	Deleted Expressed Missing	51 (65) 28 (35) 30	20 (61) 13 (39) 14	31 (67) 15 (33) 16
Stage at initial diagnosis	I II III IV Missing	27 (26) 17 (16) 38 (37) 22 (21) 5	12 (26) 8 (18) 17 (38) 8 (18) 2	15 (25) 9 (15) 21 (36) 14 (24) 3

BAP-1, BRCA1-associated protein 1; CCI, Charlson Comorbidity Index; ECOG PS, Eastern Cooperative Oncology Group performance status; IHC, immunohistochemistry; ipi-nivo, ipilimumab-nivolumab; OS, overall survival; PD-L1, programmed death-ligand 1; TPS, tumor proportion score.

A total of 21% of patients had stage IV disease at diagnosis. Meanwhile, 75% of tumors displayed epithelioid, 9% biphasic, and 16% sarcomatoid histology. Of the patients treated with 1L ipi-nivo, 60% had epithelioid MPM, whereas in the 2L+ setting, 87% had epithelioid histology. Programmed death-ligand 1 expression was available for 58 patients (53%). The distribution of PD-L1 expression according to histology is reported in Supplementary Table 1. Of those patients receiving 1L ipi-nivo, 44% (12 of 27 tested) were PD-L1 positive (tumor proportion score of ≥1%); whereas for patients with 2L+ ipi-nivo, only 29% (9 out of 31) were PD-L1 positive. BAP1 was evaluated in 72% of tumor samples and was found to be deleted in 65%.

### Patterns of Treatment and Sequencing

A total of 47 (43%) patients received ipi-nivo as 1L therapy (Supplementary Fig. 1). In those, treatment was still ongoing at data cut-off in 11 patients (23%), 13 patients (28%) subsequently received platinum-pemetrexed with or without bevacizumab, one patient carboplatin-gemcitabine, two patients pemetrexed monotherapy and one patient gemcitabine-atezolizumab in a trial. Overall, five patients were treated beyond radiographic progression with a median duration of post-progression treatment of 2.7 months (Range: 0.7–8.1). 16 patients (34%) died without any further systemic treatment after 1L ipi-nivo and three patients were still alive without having received further treatment at the time point of data cut-off.

A total of 62 patients (57%) received ipi-nivo in the second (68%) or later line (32%) setting. All these patients had previously been exposed to platinum-doublet chemotherapy (±bevacizumab) at least once. At the data cut-off, five patients (8%) remained on 2L+ ipi-nivo. Twenty-five patients (40%) died without further treatment after 2L+ ipi-nivo, 28 patients (45%) patients received further systemic therapy, mostly single-agent chemotherapy, and four patients were still alive without having received further treatment.

In the entire cohort, 30 patients (28%) had one, and 33 patients (30%) had two lines of systemic therapy, whereas almost half of the patients (42%) had three or more (up to 7 lines) of systemic treatment. Twenty-nine patients (27%) received palliative radiotherapy (RT) before or during immunotherapy (for pain control or oligoprogression).

### Efficacy Outcomes

At the data cut-off, the median follow-up for OS from the start of ipi-nivo treatment was 16.6 months (15.9 months for 1L and 16.6 months for 2L/later line). The median duration of treatment for ipi-nivo was 5.3 months when given in 1L (95% CI: 2.8–10.4) and 2.4 (95% CI: 1.9–3.3) months in 2L+ (Supplementary Fig. 2). The most common reasons to stop treatment were disease progression/death in most of the patients (62%) and toxicity in 18% of patients.

#### First Line Cohort

**Figure 1:**
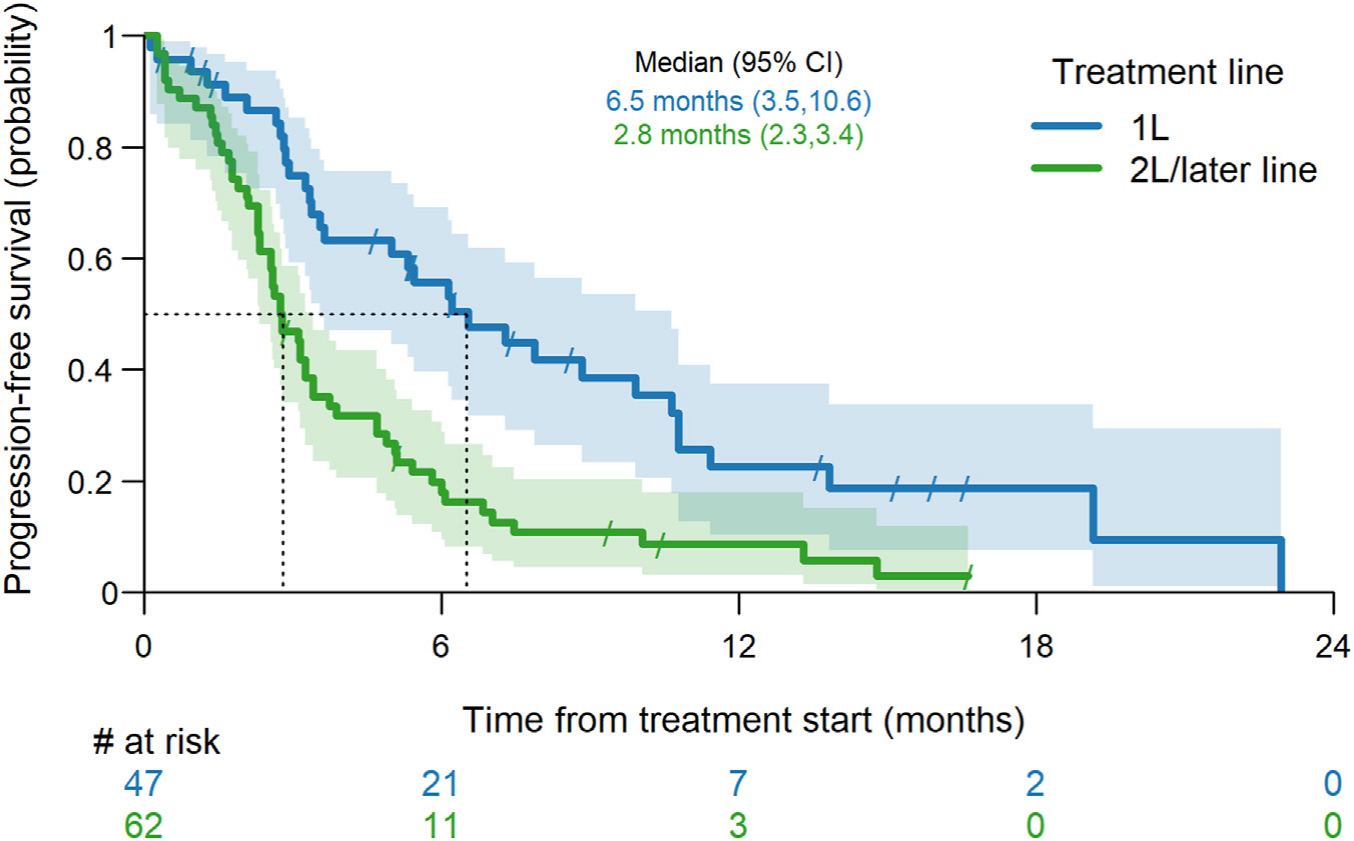
Progression-free survival by treatment line.

**Figure 2:**
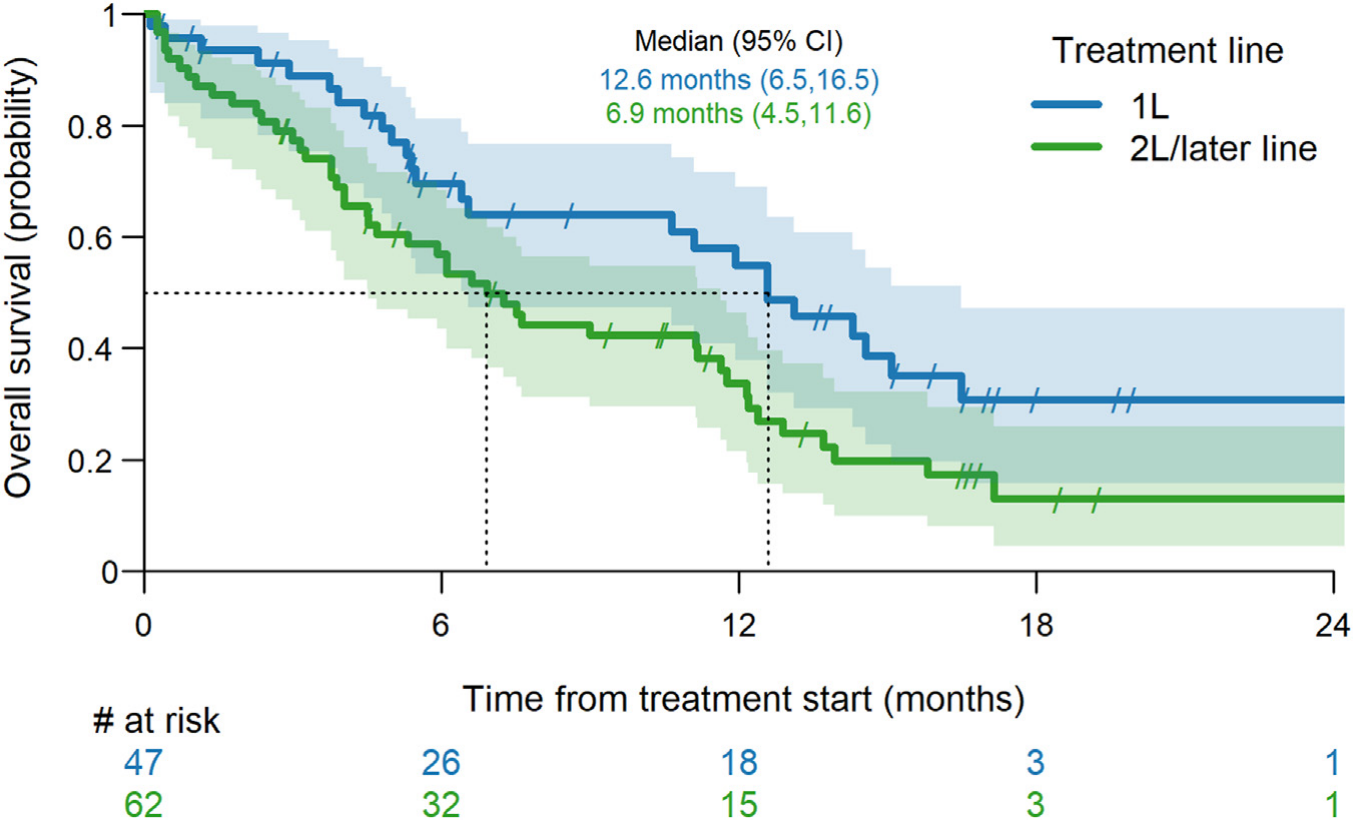
Overall survival by treatment line.

The ORR was 21% with no complete responses and the DCR was 62% whereas 28% of patients were refractory to ipi-nivo. Median PFS was 6.5 months (95% CI: 3.5–10.6) (Fig. 1) and median OS from the start of ipi-nivo was 12.6 months (95% CI: 6.5–16.5) (Fig. 2). The one-year OS rate was 55% (95%CI 38-69%) and the 18-month OS rate was 31% (95% CI: 16%–47%).

#### Second and Later Line Cohort

The ORR was 15% without any complete response and the DCR was 34%. 47% of patients were refractory to ipi-nivo. Median PFS was 2.8 months (95% CI: 2.3–3.4) (Fig. 1) and median OS after the start of 2L+ ipi-nivo was 6.9 months (95% CI: 4.5–11.6) (Fig. 2). The one-year OS rate was 34% (95% CI: 22%–46%) and the 18-month OS rate was 13% (95% CI: 5%–26%).

### Survival Outcomes in Subgroups of Interest (Both Cohorts Pooled)

Numerical differences in median PFS were seen according to histology, PD-L1 expression, and ECOG PS, but only the latter was statistically significant (1.3 months in ECOG PS ≥ 2 versus 3.9 months in patients with an ECOG PS of 0–1, *p* < 0.001). (Supplementary Table 2). Nevertheless, a trend for improved median PFS in PD-L1 positive patients was observed (2.8 months versus 6.8 months, *p* = 0.19) (Supplementary Fig. 3*A*) in a sensitivity analysis in patients with epithelioid MPM.

**Figure 3:**
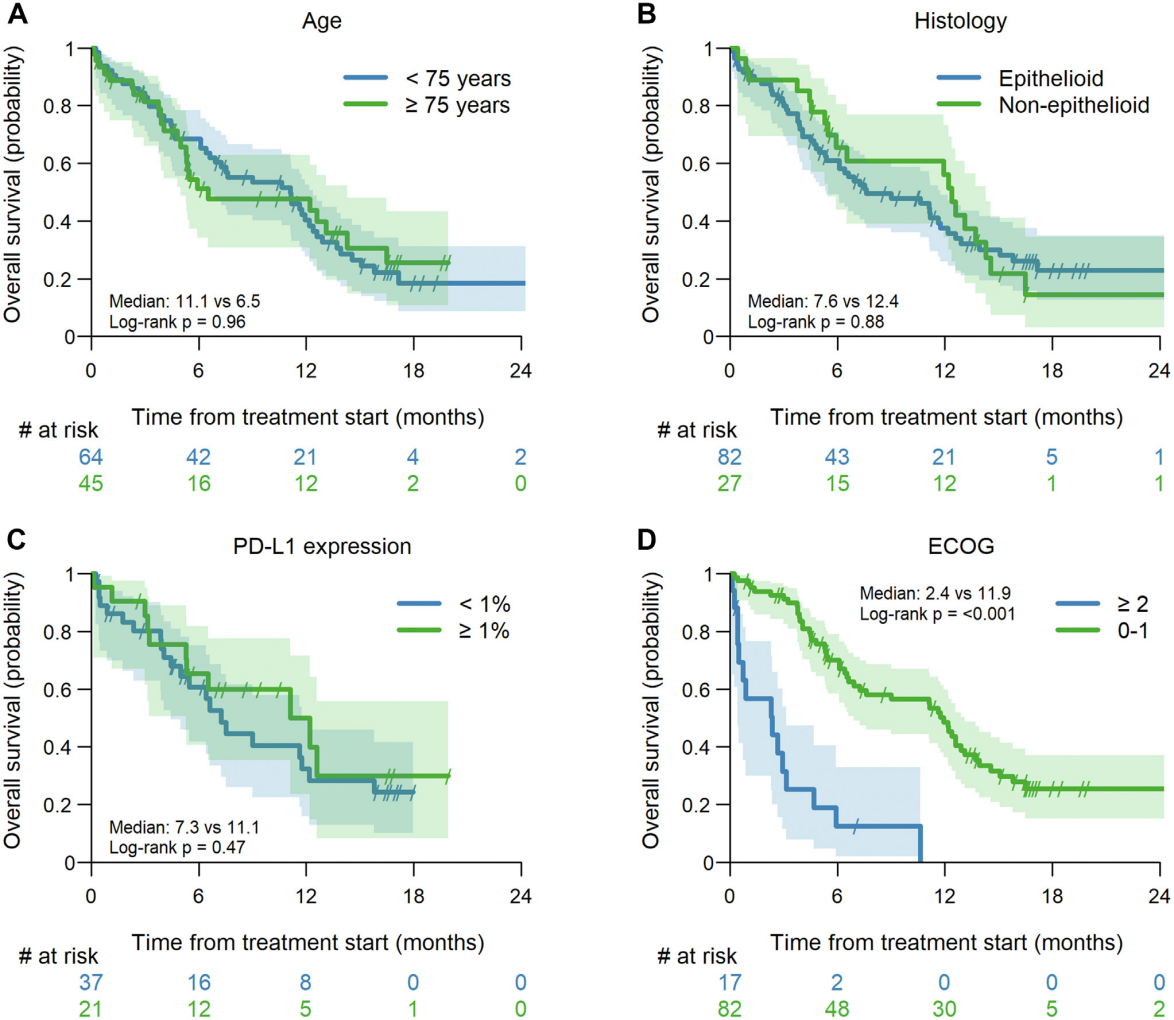
Overall survival by key subgroups of interest: (*A*) age (elderly ≥ 75 and <75 years); (*B*) histology (epithelioid versus non-epithelioid); (*C*) PD-L1 expression (< versus ≥ 1%); (*D*) ECOG performance status (≥2 versus 0–1).

Comparable to the subgroup analyses for PFS, no significant differences in OS were observed depending on age, histology, and PD-L1 expression (Figs. 3*A*–*C*) whereas patients with a poor ECOG PS of 2 or higher at the start of ipi-nivo had a significantly shorter OS as compared with patients with an ECOG PS of 0 to 1 (11.9 months versus 2.4 months, *p* < 0.001) (Fig. 3*D*). This finding was confirmed in a sensitivity analysis only looking at patients treated with 1L ipi-nivo (Supplementary Fig. 4).

In patients with epithelioid MPM and available BAP1-testing data, the median OS was 11.1 months when BAP1 expression was lost and 5.0 months with maintained BAP1 expression (*p* = 0.092) (Supplementary Fig. 5).

Twenty-nine patients (27%) had received some form of tumor irradiation during or before ipi-nivo treatment. For those with prior RT, the median time from the end of RT to ipi-nivo treatment start was 4.0 months. Patients with prior or concurrent RT had numerically longer median OS than those without prior or concurrent RT (11.9 versus 7.5 months, *p* = 0.824) (Supplementary Fig. 6).

The effect of treatment sequencing was analyzed in a subset of patients with epithelioid MPM where ipi-nivo was either given as 2L treatment after platinum-pemetrexed (±bevacizumab) (n = 35) or as 1L treatment followed by platinum-pemetrexed (±bevacizumab) (n = 8) (Supplementary Fig. 7). In these patients, we observed a trend toward improved OS for the sequence of ipi-nivo followed by chemotherapy as compared with chemotherapy followed by ipi-nivo (53.6 months versus 19.2 months, *p* = 0.35) (Supplementary Fig. 8).

### Safety

In total, 105 patients were included in the safety analysis. Data on AEs was missing in 4 patients. Adverse events related to ipi-nivo treatment (TRAEs) of any grade occurred in 65 (62%) patients. The highest-grade TRAE was G1 in 14%, G2 in 22%, G3 in 21%, G4 in 2% and G5 in 3% of the patients (Table 2). The most frequent TRAEs observed in 5% or more patients were rash (14%), colitis (10%), hepatitis (11%), infusion reactions (11%), pneumonitis (8%), arthritis (7%), pruritus (7%) and hypothyroidism (6%). A total of three fatal TRAEs occurred (one colitis and two pneumonitis). Treatment-related adverse events leading to treatment discontinuation occurred in 24 patients; in 18% of the patients with epithelioid, 0% with biphasic, and 29% with sarcomatoid MPM. Twenty patients stopped both agents, three patients only ipilimumab, and one patient only nivolumab (because of a G3 infusion-related reaction). The most frequent TRAEs of grade 2 or higher leading to discontinuation of immunotherapy were colitis (in four patients), hepatitis (in six patients,) and pneumonitis (in seven patients). Forty-one patients (39%) were treated with corticosteroids: 13 patients with intravenous, 24 patients with oral, nine patients with topical, and one with other corticosteroids (some patients received corticosteroids by more than one route). Seven patients received additional immunosuppressive treatments (such as infliximab, vedolizumab, natalizumab, methotrexate, and mycophenolate mofetil).

**Table 2:** Safety Summary

Variable	Category	Overall Cohort
Total N (%)		105^a^
Adverse events (Highest grade/patient)	No reported AEs G1 G2 G3 G4 G5	40 (38) 15 (14) 23 (22) 22 (21) 2 (2) 3 (3)
Treatment modifications due to Toxicity	No Treatment modification Treatment interruption (without discontinuation) Permanent discontinuation of Ipi only Permanent discontinuation of Nivo only Permanent discontinuation of Nivo and Ipi	71 (68) 10 (9) 3 (3) 1 (1) 20 (19)
Use of immunosuppressive agents^b^	No use Topical corticosteroids Oral corticosteroids Intravenous corticosteroids Other immunosuppressive agents	64 (61) 9 (9) 24 (23) 13 (12) 7 (7)

AEs, adverse events; G, grade; Ipi, ipilimumab; Nivo, nivolumab.

a Safety population includes only 105 patients as data on adverse events was missing in four patients included in the overall analysis.

b Some patients received corticosteroids by more than one route.

We did not observe any significant differences in baseline characteristics such as sex, age, histology, CCI, or ECOG PS between patients experiencing any grade TRAEs and those who did not (Supplementary Table 3). Nevertheless, TRAE of any grade occurred more frequently in patients with a history of atopy/allergy (TRAEs developed in 15 out of 17 patients) than those without atopy/allergy (49 out of 87 patients). The same was true for patients with a history of any auto-immune disease (AID). Six out of seven patients (86%) with a history of AID experienced a TRAE, whereas TRAEs only occurred in 59 of 98 patients (60%) without a history of AID. Importantly, the median OS in patients with ipi-nivo discontinuation due to TRAEs was similar to those without discontinuation (11.4 months versus 7.5 months, *p* = 0.664) (Supplementary Fig. 9).

## Discussion

We report on two RW cohorts of patients with MPM who received ipi-nivo either as 1L or as 2L+ treatment at 12 Swiss cancer centers. Soon after the publication of the CM743 trial results, ipi-nivo was approved in Switzerland. Although there is no approval for ipi-nivo as later-line treatment in MPM, health insurance companies and in particular the Swiss Accident Insurance Fund (the public insurance company covering health issues due to work-related asbestos exposure) have mostly been reimbursing off-label ipi-nivo in later line on the basis of the MAPS2 trial results.

The median age of patients in our registry was the same as in MAPS2^8^ and CM743.^1^ Apart from the 16% of patients who had an ECOG PS of 2—a population that was excluded in the prospective trials—the proportion of patients with PS 0 and PS 1 were similar, with only 24% of patient with PS 0, reflecting the fact that MPM occurs in an older population^11^ and is often accompanied by systemic symptoms affecting ECOG PS.^12^

Although the proportion of patients with epithelioid versus non-epithelioid MPM who received 2L+ ipi-nivo was similar in our registry as in the MAPS2 trial (approximately 85% versus 15%), we found a lower proportion of patients with epithelioid histology (60%) treated with 1L ipi-nivo than in CM743 (75%). This is related to the fact that for epithelioid MPM, the Swiss approval of ipi-nivo as 1L treatment is restricted to a PD-L1 of 1% and higher for MPM (no limitation due to PD-L1 expression in patients with non-epithelioid MPM).

The interpretation of our results with regard to PD-L1 expression is limited as local PD-L1 testing was only done in approximately 50% of the tumor samples. We noted a higher proportion (64% in the entire cohort, and 56% and 71% in the 1L and 2L+ cohorts respectively) of PD-L1 negative MPM than in MAPS2 (44%) or CM743 (20%).

In our RW cohorts, efficacy outcomes for ipi-nivo were generally inferior to those reported in the prospective trials. In the 1L setting, ORR was 21% versus 40%, median PFS 6.5 versus 6.8 months, median OS 12.6 versus 18.1 months when compared with CM743. Ipilimumab in combination with nivolumab as 2L+ treatment also yielded inferior efficacy in the RW setting as compared with the MAPS2 population with an ORR of 14.5% versus 28%, median PFS of 2.8 versus 5.6 months, and median OS of 6.9 versus 15.9 months. The higher rate of PD-L1 negative tumors—particularly in our 2L+ cohort—may explain part of the difference as the efficacy of immune-checkpoint inhibitors in mesothelioma does report some correlation with PD-L1 expression.^1^ Nevertheless, the role of PD-L1 expression as a predictive biomarker to guide treatment selection in MPM is still controversial.^13^ On the other hand this might be counterbalanced by the fact that increased PD-L1 expression is overall associated with worse prognosis in mesothelioma and has therefore recently been integrated as a parameter into the new Cancer and Leukemia Group B prognostic score.^14^ Nevertheless, in the RW setting as in our cohorts the most important reason for inferior outcomes is related to the treatment of more vulnerable patients with more comorbidities, impaired organ function, and especially patients with a poor ECOG PS of 2 or higher and to inclusion of fewer patients with an indolent course.^15^ The decrease in survival outcomes (and often response rates) when results of prospective trials (especially earlier phase trials) are transferred to the RW setting is expected and was also observed for patients with MPM receiving 1L platinum-pemetrexed treatment^16^ and later line monotherapy with anti-PD-1 antibodies.^17^ Our survival results for patients receiving 1L ipi-nivo are also in line with other recently published 1L ipi-nivo RW MPM-cohorts: one from two cancer centers in the Netherlands,^18^ the RIOMeso analysis from Australia^19^ and the German database^20^ The only RWD reporting similar survival outcomes as CM743 was the French MESOIMMUNE database from the ipi-nivo early access program^21^ where only 8% of patients had an ECOG PS of 2.

It is important to emphasize that outcomes with ipi-nivo for patients with an ECOG PS ≥ 2 were particularly poor in our cohort. Whereas the median OS in the 1L setting in patients with an ECOG PS of 0 to 1 was 14.6 months, this was significantly inferior in patients with a PS of 2 or higher with only 2.9 months and therefore represents an important prognostic factor. A similar observation was made in patients with metastatic NSCLC in the randomized phase III Energy-GFPC 06-2015 where patients with an ECOG PS of 2 may even have had a detrimental effect of 1L ipi-nivo as compared with platinum-based chemotherapy ((median OS 2.9 versus 6.1 months with chemotherapy).^22^ Therefore, in our opinion, the use of ipi-nivo in patients with mesothelioma having an ECOG PS of 2 or higher should be critically questioned and careful evaluation of treatment goals and options including the best supportive care strategy is required in these frail patients.

Although it is undebatable that 1L ipi-nivo is the preferred choice for patients with non-epithelioid MPM on the basis of the clear OS-benefit in this subpopulation in CM743, it is less clear whether ipi-nivo should be preferred as 1L treatment over platinum-pemetrexed chemotherapy (±bevacizumab) in patients with epithelioid MPM. The MAPS trial^23^ resulted in a median OS of 18.1 months in patients with epithelioid MPM receiving 1L platinum-pemetrexed-bevacizumab—exactly the same number as for patients with epithelioid MPM receiving 1L ipi-nivo in CM743. Notably, bevacizumab is not reimbursed for MPM in many countries If both options are available, the choice between 1L ipi-nivo versus 1L chemotherapy for epithelioid MPM is often on the basis of factors such as tumor burden and systemic inflammatory symptoms. In case the latter two are elevated—which often correlates with poorer ECOG PS—platin-pemetrexed (±bevacizumab) is often preferred as 1L treatment over ipi-nivo by many clinicians, a practice that is mainly driven by clinical experience and published data on the negative impact of elevated inflammatory markers such as neutrophil-leukocyte-ratio on immunotherapy treatment outcomes.^24^ Unfortunately, the European Organization for Research and Treatment of Cancer prognostic scores for MPM which take into account the systemic inflammatory score (through white blood cell count)^25^ could not be calculated in our cohort as laboratory values were not retrieved.

Half of the patients (47.9%) in the ipi-nivo arm of CM743 received at least one further treatment after progression, mainly platinum-based chemotherapy (±bevacizumab). Yet treatment outcomes for second-line platinum-based chemotherapy after ipi-nivo have not been reported so far. In our cohort, the numerically higher median OS for patients receiving 1L ipi-nivo and subsequently receiving at least one further line of treatment including platin-pemetrexed chemotherapy (n = 8, median OS = 53.6 months) versus those with ipi-nivo after chemotherapy (n = 35, median OS = 19.2 months) may have been influenced by small numbers, many censoring events and the fact that more patients had PD-L1 positive tumors in the 1L than in the 2L+ ipi-nivo cohorts. It will be interesting to compare outcomes with 1L platinum-pemetrexed-PD-(L)1 inhibitor (on the basis of the IND.227 trial results^26^) with 1L ipi-nivo in patients with epithelioid MPM, particularly in the presence of high tumor burden or systemic inflammatory markers to better understand whether the addition of chemotherapy to immunotherapy might mitigate the negative impact of elevated inflammatory markers on immunotherapy outcomes. Interestingly, the addition of atezolizumab to platinum-pemetrexed-bevacizumab did not significantly improve OS in the overall population in the BEAT-Meso trial.^27^ Overall, the sequencing strategies as reported in our RW cohorts most likely benefit more patients than combining chemo- and immunotherapy upfront when sparing superimposed toxicities.

Rates of adverse events in RW cohorts are often similar to prospective registration trials despite the fact that more vulnerable patients are included. Underreporting of AEs in registries may contribute to that observation.^15^ In our cohort a slightly higher rate of G3 to G4 TRAE was observed (42% in our overall population versus 30% in CM743 and 26% in MAPS2), whereas G5 TRAEs (3% versus 1% and 5%) and treatment discontinuation due to TRAE (19% versus 21% and 23%) occurred at similar rates. One possible reason for an increase in G3 to G4 TRAEs is that 16% of our patients had a history of atopy/allergy and 6% had pre-existing auto-immune disease (arthritis, dermatitis, psoriasis, pneumonia, or autoimmune-thyreoiditis). Indeed, a correlation between a history of atopy/allergy or autoimmune disorder and the occurrence of any grade TRAE was noted in our cohort.

Our study has several limitations. First, the retrospective design and the small number of patients limit the strength of our findings. Although numbers were small in each cohort (1L and 2L+) and their subgroups, the reporting of both cohorts in one RWD analysis allowed for comparisons and inference to optimal sequencing strategies. There was no centralized radiographic review of outcomes, modified Response Evaluation Criteria in Solid Tumors criteria for mesothelioma were not consistently used in all contributing centers and PFS was assessed by follow-up imaging at intervals chosen by the treating physicians, as it is usually the case in a RW setting. A history of atopy/allergy was attributed by the physician with no strict definition prespecified and data on the nature and severity of atopy/allergy was not collected. Furthermore, PD-L1 expression was also only available for a subset of patients and was assessed locally. In this regard, translational research on centrally assessed tumor samples with the aim of further elucidating proteomic, genetic, and transcriptional markers correlated to outcome is ongoing.

## Conclusion

In summary, we report on the outcomes of ipi-nivo in patients with MPM treated in routine clinical care in Switzerland and note inferior efficacy but comparable safety results to the prospective trials. At the same time, the particular benefit of combination immunotherapy in patients with non-epithelioid mesothelioma is confirmed. Ipi-nivo should therefore remain the preferred 1L treatment choice for non-epithelioid MPM, whereas equipoise exists for epithelioid MPM. ECOG PS of 2 or higher represents a relevant poor prognostic factor also with ipi-nivo, questioning use of this regimen in this frail population.

## Ethics Statement

All procedures performed in studies involving human participants were in accordance with the ethical standards of the local research committee and with the 1964 Helsinki declaration and its later amendments or comparable ethical standards. The local research ethics board has approved this research (BASEC Nr. 2022-01537). Informed consent was not required for this study because of the retrospective data collection and the existence of a general consent for most patients.

## CRediT Authorship Contribution Statement

**Sabine Schmid:** Conceptualization, Methodology, Investigation, Resources, Data curation, Writing - original draft, Writing - review and editing, Visualization, Supervision, Project administration, Funding acquisition.

**Lisa Holer:** Methodology, Formal analysis, Resources, Data curation and visualization, Writing - original draft, Writing - review and editing.

**Katrin Gysel:** Methodology, Formal analysis, Resources, Data curation and visualization, Writing - original draft, Writing - review and editing.

**Kira-Lee Koster:** Investigation, Resources, Writing - review and editing.

**Sacha Rothschild:** Investigation, Resources, Writing - review and editing.

**Laura A. Boos:** Investigation, Resources, Writing - review and editing.

**Lorenz Frehner:** Investigation, Resources, Writing - review and editing.

**Sabine Cardoso Almeida:** Investigation, Resources, Writing - review and editing.

**Christian Brithschgi:** Investigation, Resources, Writing - review and editing.

**Yannis Metaxas:** Investigation, Resources, Writing - review and editing.

**Michael Mark:** Investigation, Resources, Writing - review and editing.

**Patrizia Froesch:** Investigation, Resources, Writing - review and editing.

**Wolf-Dieter Janthur:** Investigation, Resources, Writing - review and editing.

**Anna Allemann:** Investigation, Resources, Writing - review and editing.

**Christine Waibel:** Investigation, Resources, Writing - review and editing.

**Catherine Von der Mühll-Schill:** Investigation, Resources, Writing - review and editing.

**Martin Früh:** Conceptualization, Methodology, Investigation, Resources, Writing - review and editing, Supervision.

**Laetitia A. Mauti:** Conceptualization, Methodology, Investigation, Resources, Data curation, Writing - original draft, Writing - review and editing, Visualization, Supervision, Project administration, Funding acquisition.

## Disclosure

Dr. Schmid received institutional grants from Janssen, BMS, MSD, Marlies-Schwegler Stiftung, von Tobel Stiftung, and Swiss Life and holds positions on advisory boards and institutional speaker invitations from BMS, MSD, Roche, Merck, Sanofi, Janssen, AstraZeneca, Takeda; Travel support: Roche, Takeda, Amgen. Dr. Koster received institutional travel support from Takeda, Janssen. Dr. Rothschild received institutional honoraria from Roche, Astra Zeneca, BMS, Boehringer Ingelheim, MSD, Novartis, Amgen, Lilly, Eisai, Merck, Pfizer, Takeda, Bayer, Janssen, Otsuka, Pharmamar, and Sanofi, holds institutional advisory roles in Astra Zeneca, Boehringer Ingelheim, BMS, Pfizer, Eisai, Lilly, Merck, MSD, Novartis, Roche, Takeda, Amgen, Otsuka, Pharmamar, holds positions on institutional speaker bureaus of Roche, Sanofi, Amgen, Astra Zeneca, Takeda, received institutional research funding from Abbvie, BMS, Astra Zeneca, Boehringer Ingelheim, Merck, Roche; Travel support: Sanofi, Roche, BMS, MSD, Astra Zeneca, Takeda, Boehringer Ingelheim, Amgen, and has other relationships with Federal Drug Commission of the Federal Office of Public Health, SAKK. Dr. Brithschgi holds advisory roles in Astra Zeneca, Pfizer, Roche, Takeda, Janssen, Boehringer Ingelheim, Merck, and Sanofi, and received research funding from Bayer and travel support from Astra Zeneca, Takeda, and Amgen. Dr. Mark received consulting fees from Amgen, Astra Zeneca, BMS, MSD, Pfizer, Takeda, and Roche and travel support from Astra Zeneca, Roche, and Takeda. Dr. Froesch holds personal advisory roles in BMS, Roche, Sanofi, and Takeda, and an institutional advisory role in MSD and is an invited speaker (personal) of Janssen. Dr. Janthur received travel support from 10.13039/100004337Roche and holds advisory roles in Pharmamar and MSD. Dr. Allemann received institutional honoraria from Janssen and Astra Zeneca, and travel support from Janssen and holds institutional advisory roles in Janssen and Merck. Dr. Früh holds positions on institutional advisory boards of BMS, MSD, Astra Zeneca, Boehringer Ingelheim, Roche, Takeda, Pfizer, Janssen, Daiichi-Sankyo, and Pharmamar and received unrestricted institutional grants from BMS and Astra Zeneca. Dr. Mauti holds advisory roles in Takeda, BMS, MSD, Merck, Sanofi, Novartis, AstraZeneca, Pfizer, Regeneron, Daiichi, and Sanofi, has participated in invited talks of Amgen, and received travel support from AstraZeneca, Roche, and Sanofi and research funding from Gilead and AstraZeneca. The remaining authors declare no conflict of interest.
