# Supplementary material for: Real-World Outcomes of Patients With Malignant Pleural Mesothelioma Receiving a Combination of Ipilimumab and Nivolumab as First- or Later-Line Treatment
Source: JTO Clin Res Rep. 2024 Oct 15;5(12):100735. doi: 10.1016/j.jtocrr.2024.100735 (PMC11609637; doi:10.1016/j.jtocrr.2024.100735)

**Supplementary Material:**

**Supplementary methods:**

Definition of endpoints: PFS is defined as time from treatment start of ipi/nivo to disease progression, start of local or other systemic treatment due to local or systemic progression or death. Patients without an event were censored at the date of the start of another treatment, if any, or at the date they were last known alive. PFS was also recorded for all other systemic treatments received. OS was evaluated as time from start of ipi/nivo treatment to death and separately from start of first line treatment and from diagnosis to death. Duration of response (DOR) was defined as time from first documentation of a response (CR or PR) to progression, start of local or new systemic treatment due to local or systemic progression or death. Patients without an event were censored at the date of the start of another treatment, if any, or at the date when they were last known alive. Disease control rate (DCR) was defined as percentage of patients with partial response (PR) or stable disease (SD) as best response to treatment. Treatment beyond progression was defined using a 21-day cut off (treatment continued at least > 21 days after radiographic disease progression).

**Supplementary Tables**:

**Added new Supplementary Table 1**:

PD-L1 expression according to histological subtype

| Variable | Category | Epitheloid | Biphasic | Sarcomatoid |
| --- | --- | --- | --- | --- |
| Total N (%) |  | 82 (100) | 10 (100) | 17 (100) |
| PD-L1 (TPS) | < 1%  ≥ 1%  Missing | 32 (67) 16 (33) 34 | 5 (100) 0 (0) 5 | 0 (0) 5 (100) 12 |

**Supplementary Table 2: PFS according to subgroups (including 1L and later line patients)**

| Variable | Category | Total  N=109 (%) | Events | Median PFS in months (95%CI) | P-value |
| --- | --- | --- | --- | --- | --- |
| Age at start ipi/nivo | < 75 years  ≥75 years | 64 (59)  45 (41) | 58  32 | 3.3 (2.8-5.1)  3.9 (2.9-6.5) | 0.421 |
| ECOG PS at start ipi/nivo | 0-1  ≥2  Missing | 82 (83)  17 (17)  10 | 66  16 | 3.9 (3.2-5.8)  1.3 (0.4-2.9) | **<0.001** |
| CCI | <6  ≥6 | 51 (47%)  58 (53%) | 48  42 | 2.9 (2.6, 4.7)  3.9 (3.3, 6.5) | 0.074 |
| Histology | Epithelioid  Non-epithelioid | 82 (75%)  27 (25%) | 68  22 | 3.4 (2.8, 5.0)  4.7 (2.8, 7.3) | 0.582 |
| PD-L1 | <1%  ≥1%  Missing | 37 (64)  21 (36)  51 | 27  17 | 2.9 (2.6, 5.3)  6.5 (3.2, 7.5) | 0.142 |
| Histology = Epithelioid and PD-L1 | <1%  ≥1%  Missing | 32 (67)  16 (33)  34 | 24  12 | 2.8 (2.3, 5.3)  6.8 (2.3, 7.9) | 0.189 |

**Supplementary Table 3: Baseline characteristics in patients experiencing TRAEs versus those who did not**

| Covariate | Category | Overall cohort | No TRAEs | Any grade TRAEs | P-value |
| --- | --- | --- | --- | --- | --- |
| Total N (%) |  | 105 | 40 | 65 |  |
| Age at start ipi/nivo | Median (range) | 72.0 (55-93) | 72.5 (55-84) | 72.0 (57-93) | 0.500 |
| Age at start ipi/nivo | < 75 years  ≥75 years | 63 (60)  42 (40) | 25 (63)  15 (37) | 38 (59)  27 (41) | 0.838 |
| Sex | Female  Male | 9 (9)  96 (91) | 6 (15)  34 (85) | 3 (5)  62 (95) | 0.081 |
| ECOG PS at start ipi/nivo | 0  1  ≥2  Missing | 24 (25)  57 (59)  15 (16)  9 | 6 (17)  18 (52)  11 (31)  5 | 18 (30)  39 (64)  4 (6)  4 | **0.003** |
| CCI | <6  ≥6 | 50 (48)  55 (52) | 20 (50)  20 (50) | 30 (46)  35 (54) | 0.841 |
| Histology | Epithelioid  Biphasic  Sarcomatoid | 79 (75)  9 (9)  17 (16) | 32 (80)  2 (5)  6 (15) | 47 (72)  7 (11)  11 (17) | 0.617 |
| History of atopy/allergies | No  Yes  Missing | 87 (84)  17 (16)  1 | 38 (95)  2 (5)  0 | 49 (76.6)  15 (23.4)  1 | **0.014** |
| History of auto-immune disease | No  Yes* | 98 (93)  7 (7) | 39 (98)  1 (2) | 59 (91)  6 (9) | 0.248 |

* Rheumatoid arthritis, Psoriasis, auto-immune thyroiditis, organizing pneumonia

**Supplementary Figures**:

**Supplementary Figure 1: Swimmer plot of treatment patterns/sequencing**


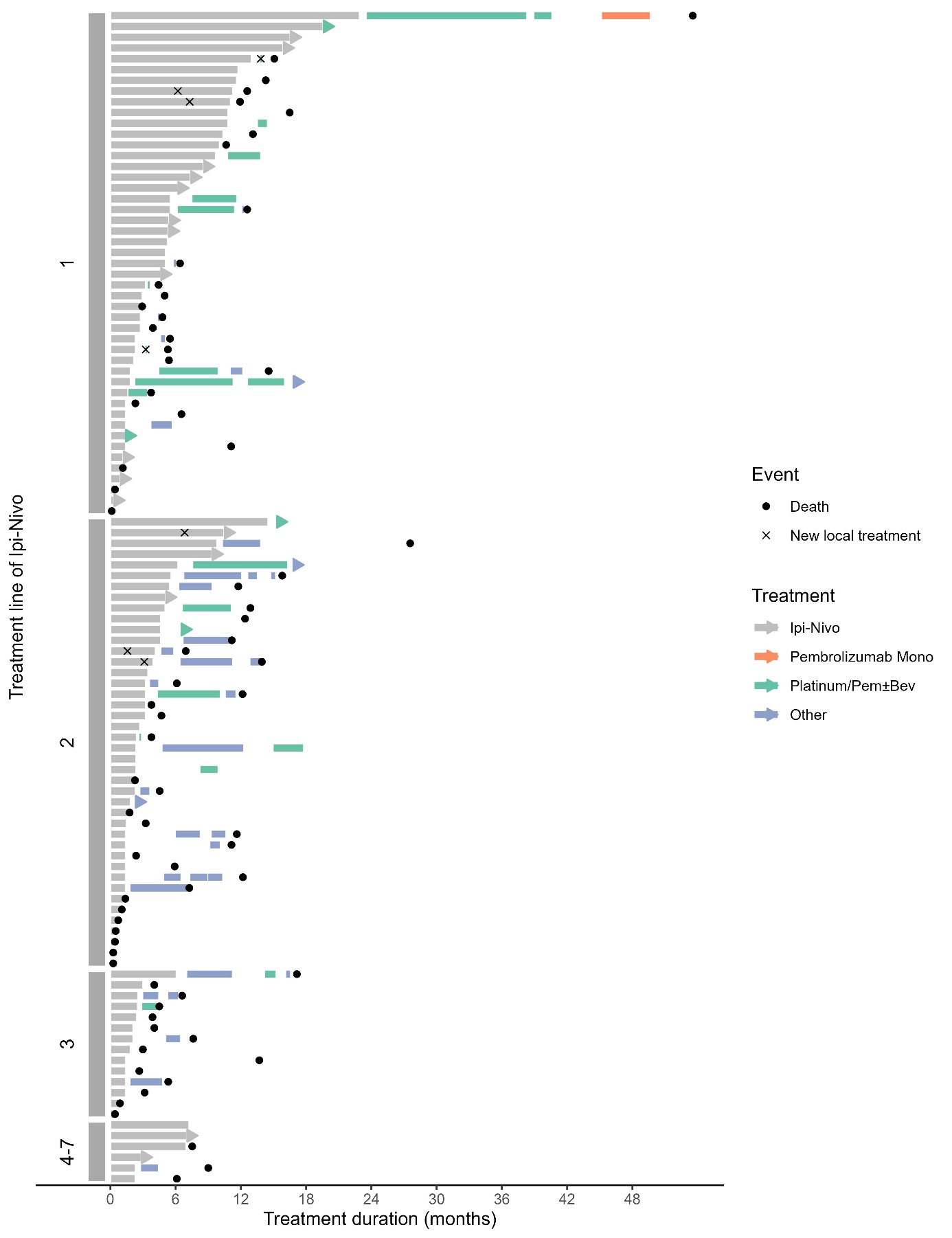


**Supplementary figure 2: Treatment duration according to treatment line**


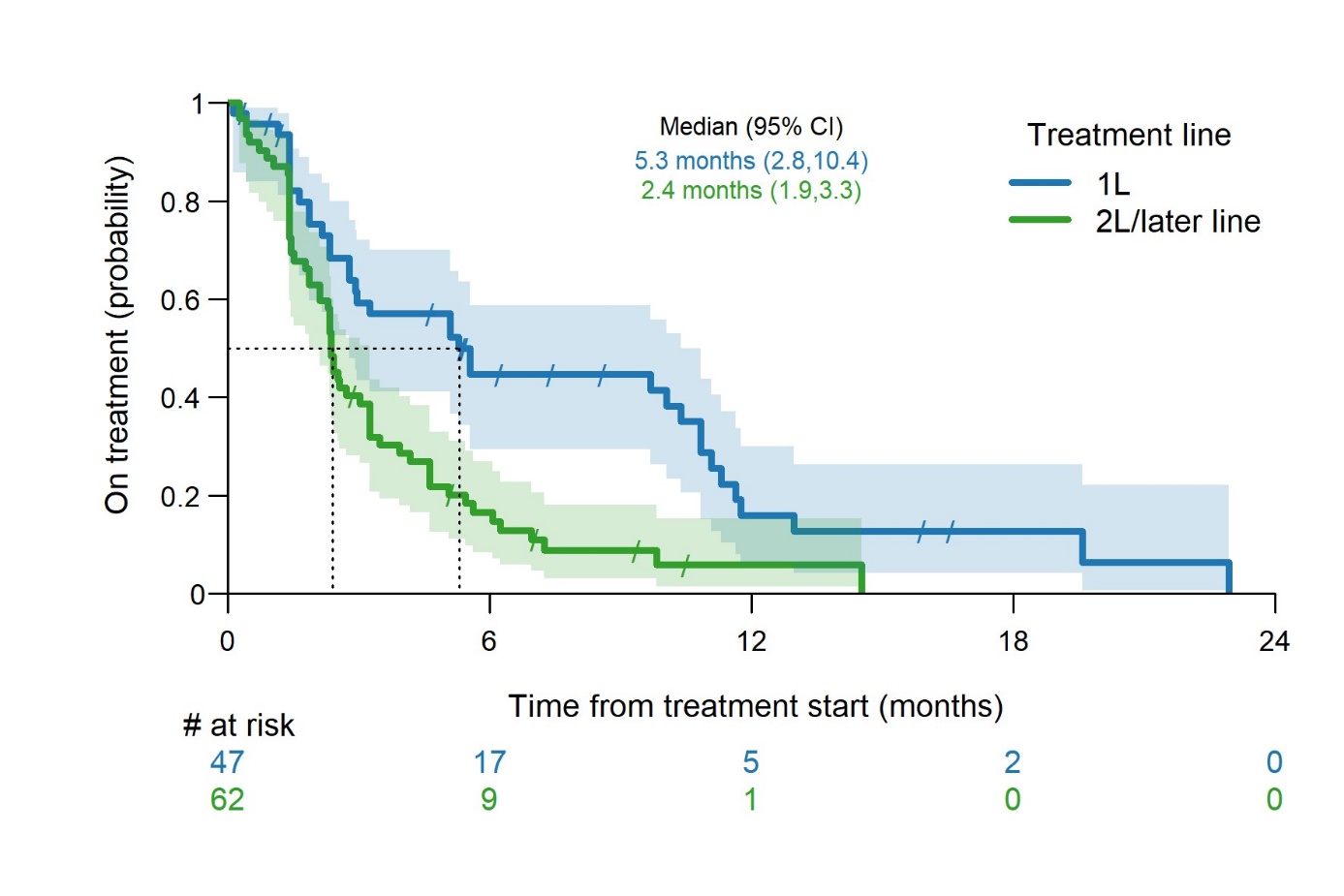


**Supplementary Figure 3: PFS according to PD-L1 expression in patients with epithelioid MPM (any line of treatment)**


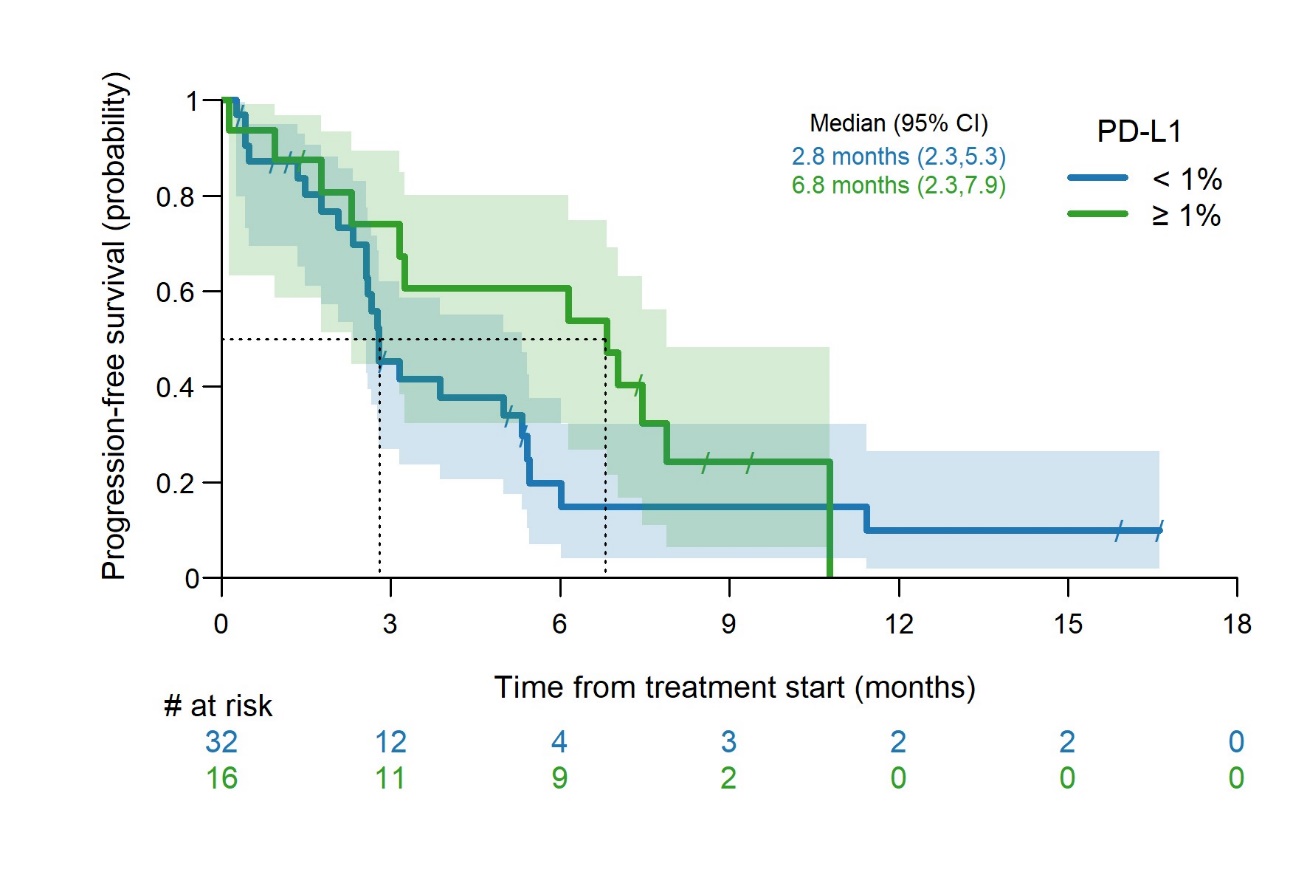


**Supplementary Figure 4: Sensitivity analysis of OS for subgroups in 1L ipi/nivo patients only**


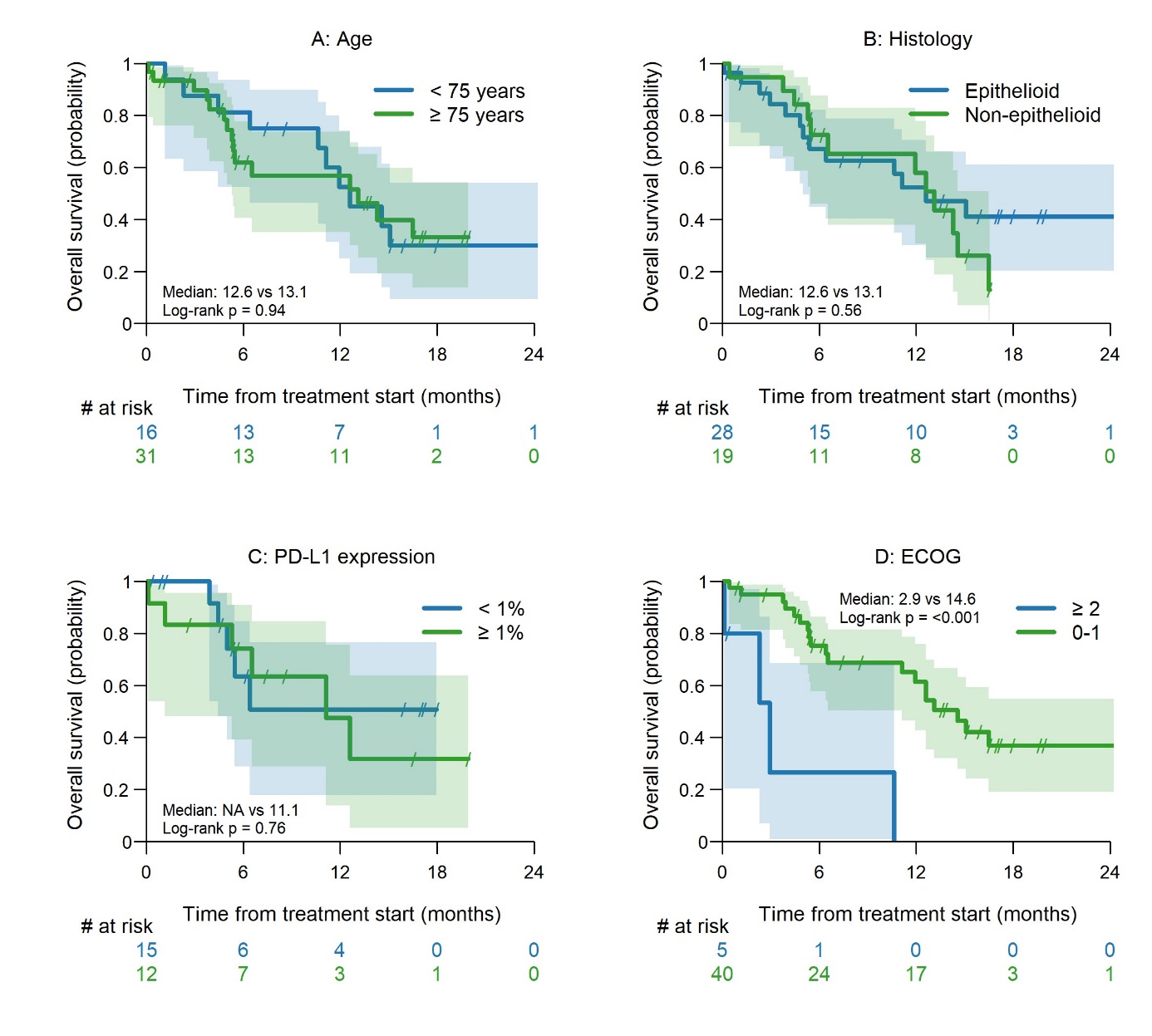


**Supplementary Figure 5: Median OS in patients with epitheloid MPM depending on PD-L1 and BAP1 expression**

**(Figure only includes patients with epithelioid MPM and available data for PD-L1 and BAP1)**


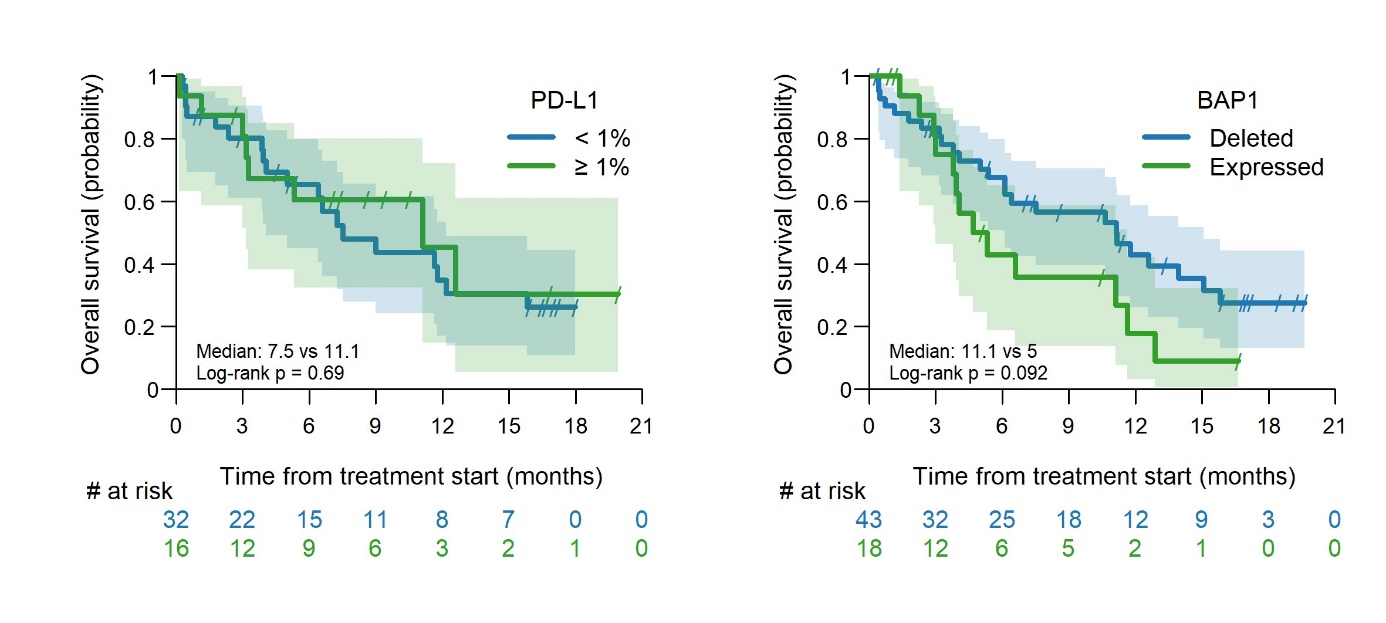


**Supplementary Figure 6: OS depending on RT prior to/during ipi/nivo**


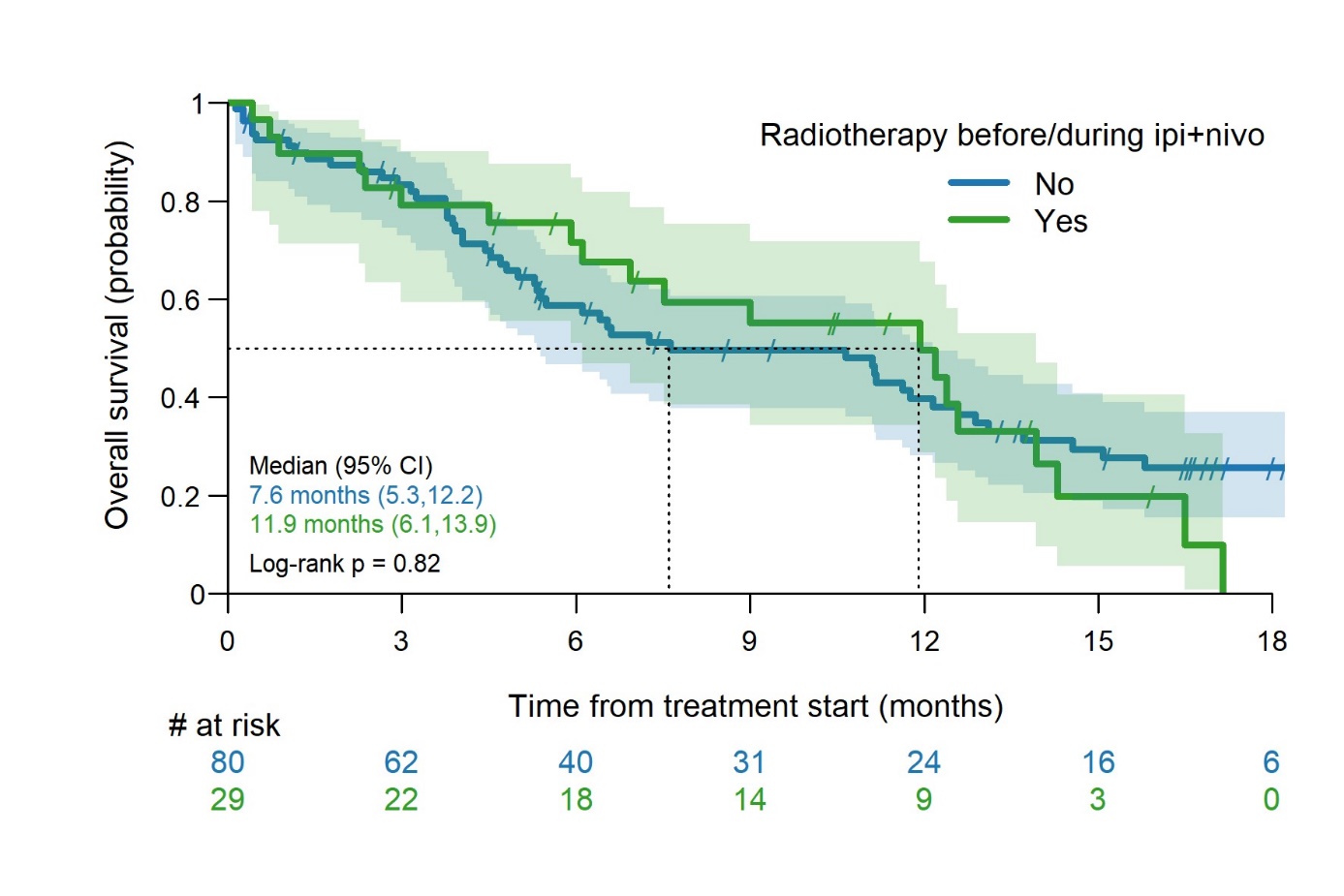


**Supplementary Figure 7: Swimmer plot illustrating treatment in patients with epithelioid MPM who were treated with the sequence ipi/nivo->platinum-based chemo or platinum-based chemo->ipi/nivo**


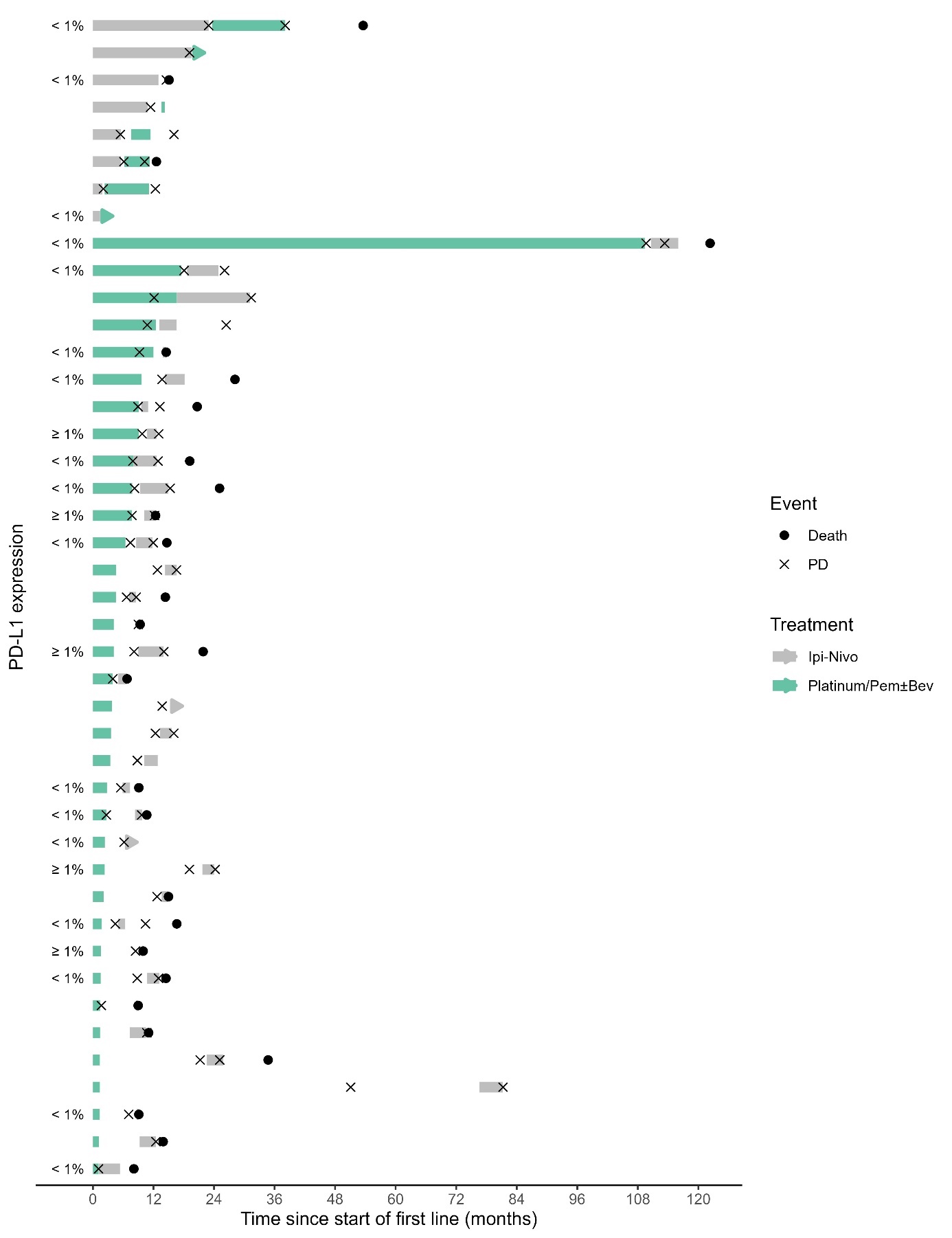


**Supplementary Figure 8: OS depending on treatment sequence (ipi/nivo->chemo versus chemo-> ipi/nivo) in epithelioid Meso**


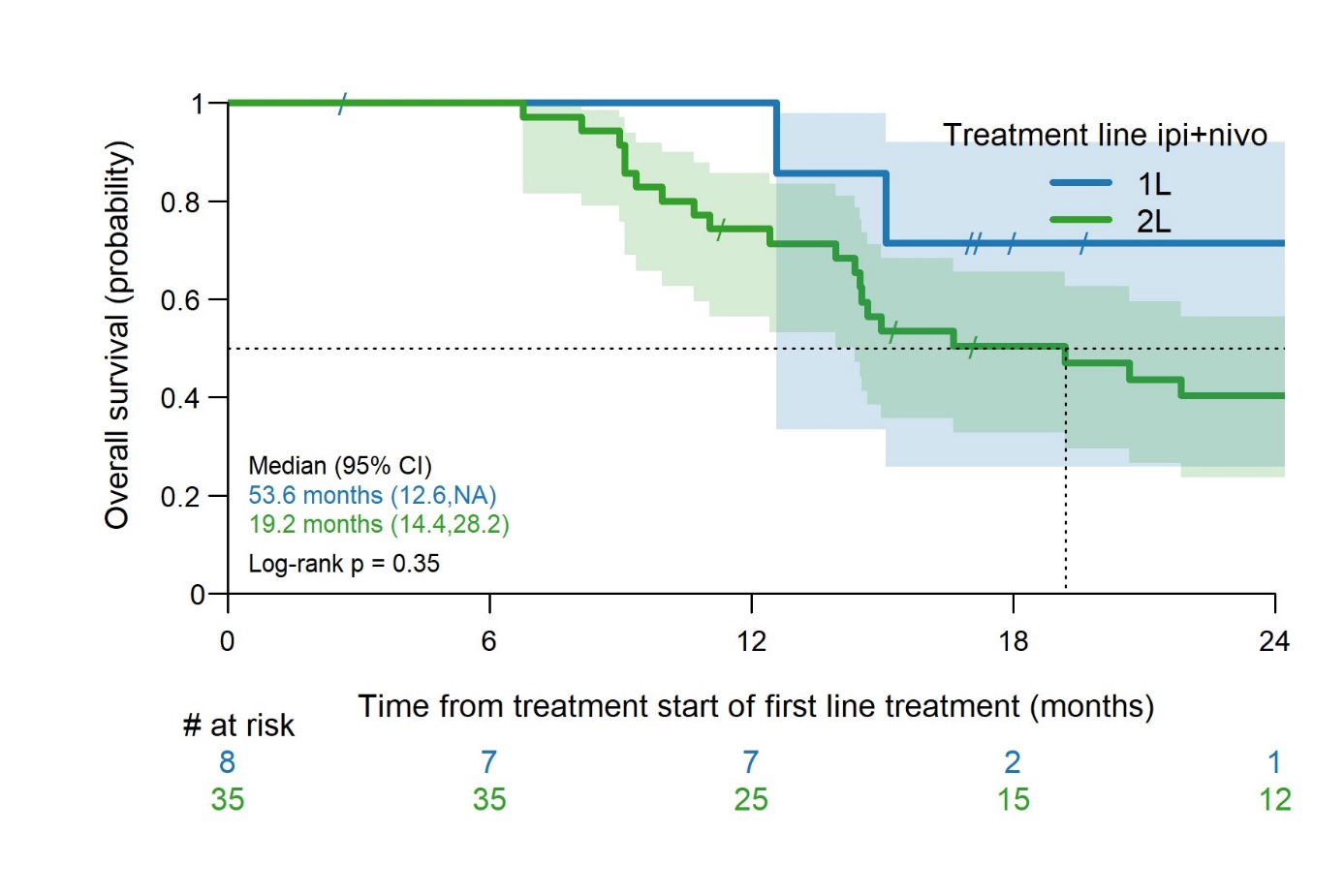


**Supplementary Figure 9: Survival in patients stopping ipi/nivo due to TRAEs versus patients without discontinuation of ipi/nivo due to TRAEs**


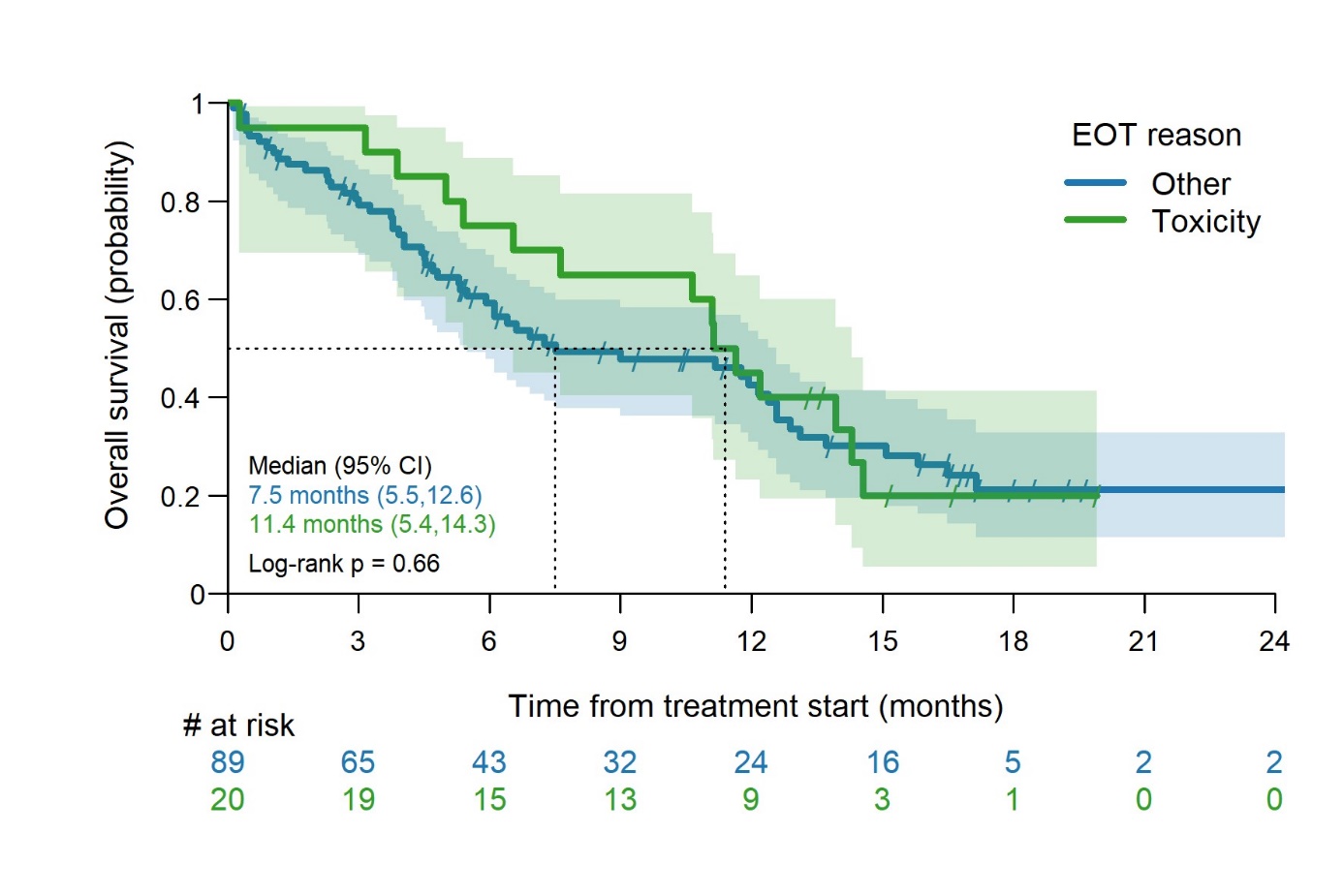

Supplement: Supplementary Material [file mmc1.docx]
